# Dysrhythmia: a specific congenital rhythm perception deficit

**DOI:** 10.3389/fpsyg.2014.00018

**Published:** 2014-02-05

**Authors:** Jacques Launay, Manon Grube, Lauren Stewart

**Affiliations:** ^1^Department of Experimental Psychology, University of Oxford Oxford, UK; ^2^Auditory Group, Institute of Neuroscience, The Medical School, Newcastle UniversityNewcastle-upon-Tyne, UK; ^3^Goldsmiths College, University of LondonLondon, UK

**Keywords:** rhythm, meter, beat, motor timing, amusia

## Abstract

Why do some people have problems “feeling the beat”? Here we investigate participants with congenital impairments in musical rhythm perception and production. A web-based version of the Montreal Battery of Evaluation of Amusia was used to screen for difficulties with rhythmic processing in a large sample and we identified three “dysrhythmic” individuals who scored below cut-off for the rhythm subtest, but not the pitch-based subtests. Follow-up testing in the laboratory was conducted to characterize the nature of both rhythm perception and production deficits in these dysrhythmic individuals. We found that they differed from control participants when required to synchronize their tapping to an external stimulus with a metrical pulse, but not when required to tap spontaneously (with no external stimulus) or to tap in time to an isochronous stimulus. Dysrhythmics exhibited a general tendency to tap at half the expected tempo when asked to synchronize to the beat of strongly metrical rhythms. These results suggest that the individuals studied here did not have motor production problems, but suffer from a selective rhythm perception deficit that influences the ability to entrain to metrical rhythms.

## INTRODUCTION

Rhythm perception and entrainment abilities develop early in human life (Hannon and Trehub, 2005; Phillips-Silver and Trainor, 2005; Zentner and Eerola, 2010) and have been suggested to be relevant to a range of functions, including mother–infant communication (Beebe and Lachmann, 1988; Bernieri et al., 1988; Dissanayake, 2000), speech and language development (Smith et al., 1989; Jusczyk et al., 1992, 1999; Huss et al., 2011; Grube et al., 2012), and social bonding (Knoblich et al., 2011). The entrainment of our body movements to a regular beat enables us to synchronize with the movements of others – a phenomenon which is argued to foster empathy (Hove and Risen, 2009; Wiltermuth and Heath, 2009; Launay et al., 2013) and can have physiological effects such as co-ordinating bodily rhythms with those of others (Konvalinka et al., 2012; Vickhoff et al., 2013) or facilitating organized motor behavior in neurological patients with movement disorders (Thaut and Abiru, 2010).

The ability to perceive and entrain to a regular beat is considered to be a common and highly automatized human ability that can be measured using perceptual and motor tasks, both behaviourally and with neuroimaging techniques (Praamstra et al., 2003; Repp, 2005; Oullier et al., 2008). There has been recent interest in the identification and investigation of individuals who appear to have difficulty with these abilities, but knowledge remains predominantly anecdotal. The few existing scientific attempts to characterize congenital rhythm impairments are typically based on motor measures (e.g., Iversen and Patel, 2008; Phillips-Silver et al., 2011; Farrugia et al., 2012; Sowinski and Dalla Bella, 2013) and more research is required to investigate these kinds of difficulties.

The most simple form of entrainment to a sensory (typically auditory) rhythmic stimulus involves perceiving and synchronizing movements with an isochronous beat with one level of periodicity, such as that produced by a metronome (Wing and Kristofferson, 1973; ten Hoopen et al., 1994; Friberg and Sundberg, 1995; Ehrle and Samson, 2005). Individuals are further able to extract the underlying beat from more complex rhythmic structures with a metrical hierarchy comprised of two or more levels of periodicities that are multiples of each other (London, 2004), and typically tend to tap along at a level corresponding to a preferred periodicity of 400 to 800 milliseconds, peaking around 600 ms (Fraisse, 1963).

The induction of a perceived beat, or meter, in the listener by the use of temporal cues alone (i.e., in the absence of changes in pitch or sound intensity) has been demonstrated to rely upon certain phenomenal principles. Phenomenal accents are found to occur on tones that are followed or preceded by a relatively long “pause,” i.e., time between consecutive event onsets or inter-onset-intervals. The feeling of a meter can be induced by rather simple rhythmic sequences of identical tones, if those phenomenal accents occur regularly at a multiple of the underlying beat unit, for instance on every fourth beat (Povel and Essens, 1985). In contrast to such strongly metrical sequences, weakly metrical ones in which accented tones do not occur at regular intervals will not induce the “feeling of a beat” in a naïve listener. A number of psychophysical studies have demonstrated an improvement in objective measures of perceptual accuracy and the subjectively perceived “feeling of the beat” or “catchiness” for metrically strong compared to weak sequences (Povel and Essens, 1985; Hirsh et al., 1990; Monahan and Hirsh, 1990; Handel, 1998; Hebert and Cuddy, 2002; Grube and Griffiths, 2009). Similarly, performance in synchronization tasks is more accurate for strongly metrical sequences compared to those that are weakly metrical (Patel et al., 2005). Whilst the ability to extract a beat from a complex auditory stimulus has been demonstrated repeatedly in groups of typically developing subjects, there has been little systematic investigation into congenital deficits in beat extraction and entrainment for such stimuli.

Individuals with a developmental disorder termed “congenital amusia,” or “tone deafness,” are characterized by deficits in the perception of pitch-related features in musical melodies, which may or may not be accompanied by deficits in the perception of rhythm-related features. The diagnostic tool, the Montreal Battery of Evaluation of Amusia (MBEA; Ayotte et al., 2002; Peretz et al., 2003) was developed as a formal test of congenital amusia. Using short musical melodies, the MBEA looks at six separate aspects of music perception including both pitch and rhythm perception tests. Peretz et al. (2003) noted that problems with pitch perception were universal in their cohort of amusic subjects, while rhythm perception was only affected in a subsample of the group. Subsequent studies have further supported the notion that congenital amusia is typically a selective pitch impairment in which rhythm deficits, where present, reflect concomitant effects of the primary pitch deficit (e.g., Peretz and Hyde, 2003; Hyde and Peretz, 2004). In a study specifically testing this hypothesis, pitch-based amusic subjects were shown to perform poorly also in rhythm discrimination tasks, but only for stimuli containing pitch variations (Foxton et al., 2006). Similarly, individuals with amusia have been demonstrated to have problems finding the beat in a musical context: when asked to move in time with musical sounds they would tap at half the expected meter (Dalla Bella and Peretz, 2003). However, the characterization of congenital amusia as a disorder of pitch, rather than rhythm, may partly reflect a screening bias, since individuals sought for such studies have typically been recruited on the basis that they self-report as “tone-deaf” and have trouble singing in tune.

Efforts to seek out people whose predominant difficulty lies in keeping in time, be it in conjunction with pitch deficits or in isolation, have been few and far between. A recent study reported on individuals who had problems with synchronization but not with rhythm perception (Sowinski and Dalla Bella, 2013). Phillips-Silver et al. (2011) investigated one particular “beat-deaf” case (“Mathieu”), who complained about an inability to find the beat in music and exhibited specific difficulties with the meter-identification task of the MBEA and in synchronizing his dance movements with a musical beat (meringue) but not with a metronome (Phillips-Silver et al., 2011). However, this study did not include a systematic investigation of metrical-beat extraction and motor synchronization with controlled auditory stimuli rather than music, leaving questions about the precise locus of the deficit unanswered.

In the present study we sought out individuals who exhibited specific impairments in rhythm perception according to the MBEA (administered on-line) and also self-reported difficulties with rhythm in everyday life. These individuals, whom we subsequently refer to as “dysrhythmic” were then tested to assess their ability to produce an isochronous tapping pulse (i) spontaneously, (ii) in time to isochronous stimulus sequences, and (iii) to sequences with strongly and weakly metrical-beats. In order to investigate the impact of pitch on these synchronization abilities, all three types of sequence were presented with and without pitch variation.

## MATERIALS AND METHODS

### PARTICIPANTS

The MBEA was used to identify individuals with impairments that were specific to the rhythm subtest of the MBEA and associated with normal performance on the pitch-related subtests (scale, contour, interval). An online version of the scale and rhythm subtests was taken by 89,000 participants, and individuals scoring below the published cut-off scores for the rhythm subtest and above the cut-off score for the scale test (Peretz et al., 2003) were identified. Individuals scoring in this way were asked to retake these two online tests. Provided they continued to score below and above cut-off for the rhythm and scale subtests respectively and they self-reported as having difficulties with rhythm in everyday life, they were invited to the laboratory for testing on four of the MBEA subtests (scale, contour, interval, and rhythm) to verify that their profile of scores was robust. Three individuals were identified: SS, female, aged 26; SWI, male, aged 43; and VPO, female, aged 29 and included in the main study.

Thirty eight control participants (18M, 20F; age range 19–61, mean = 38.92) were also identified from the online database for further inclusion in the present study. These individuals had scored above the published cut-off scores, both for the online MBEA subtests (scale and rhythm) as well as during laboratory-based testing (scale, contour, interval, rhythm subtests). In the tapping tasks, data for two dysrhythmic participants were not recorded for one trial due to a technical error; similarly, data were not recorded for eight trials in one control participant so that particular participant’s data are not included in any synchronization task analysis. All participants were reimbursed for travel expenses, and additionally received £7.50 for their time (approximately one hour).

### EQUIPMENT

All tapping tasks were performed using a DELL XPS M1530 computer running MAX/MSP 4.5 software. Stimuli were played to participants using an external Alesis IO2 soundcard and Sennheiser HD 265-1 headphones. Participants tapped on the computer keyboard using their preferred hand.

### STIMULI

**FIGURE 1:**
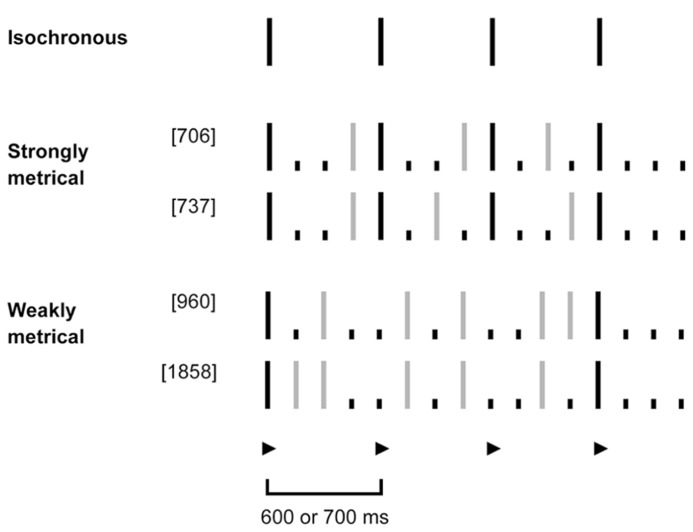
**Schematic representation of tapping stimuli: isochronous, strongly and weakly metrical tone sequences.** Isochronous sequences consisted of evenly spaced tones, occurring every 600 or 700 ms (depicted in black). For the metrical sequences, the underlying meter with a beat of 4 had a corresponding period of 600 or 700 ms, respectively. The beat locations (unit 1, 5, 9, 13) are denoted by a ▶. In order to induce the feeling of a metrical-beat of 4 in the listener, the strong sequences had a phenomenally (purely due to temporal spacing) accented tone on all four intended downbeat locations (depicted in black). The weak sequences in contrast had an accented tone only on the first and last downbeat (in black), but no tones on the second and third. Gray lines denote unaccented tones, dots silent units.

The strongly and weakly metrical rhythms used in the tapping tasks were of the same type as those developed by Grube and Griffiths (2009), following the phenomenally based rules of metrical-beat induction described by Povel and Essens (1985). Strongly metrical rhythms had accented tones on all four downbeat locations for a meter of 4 (i.e., units 1, 5, 9, 13 of 16) while weakly metrical rhythms had accented tones in only two of those hypothetical downbeat locations (first and last) and two silent beat locations in-between (second and third). Both sets of rhythms have a larger number of silent units than would be required to induce a meter of 2 or 3, so that a meter of 4 was the most likely to be perceived based on the model of Povel and Essens (1985). The important difference between these two sets of rhythms is the strength of meter that they induce. While the strongly metrical rhythms convey a clear sense of the metrical beat, this is very hard to find in the weakly metrical rhythms. The sequences used had the same number of tones and accented beats, were composed of the same intervals, and featured the same overall meter to control for any other influences on complexity of rhythm. They corresponded to sequence numbers 706, 737, 960, and 1858 as reported by Grube and Griffiths (2009) and are depicted in **Figure 1**.

Across all synchronization trials, the underlying tempo of rhythms was varied between 600ms and 700ms – both of these have been deemed to be comfortable tapping rates (Repp, 2005) and no difference in synchronization accuracy or tapping variability was expected between these two tempi. The variation in tempo was introduced so that people would not entrain with the isochronous sequences presented first and continue tapping at this rate throughout the other trials. The starting tempo was counterbalanced between participants.

Random pitch variation was introduced throughout half of the trials to determine whether dysrhythmic participants experience further distraction given a pitch variation or not. Each sequence was presented once at a constant pitch and once with the random pitch variation. Random pitch variation was generated during the task and notes could take any semitone value within a two-octave scale. A full list of trial types is given in **Table 1**.

**Table 1:** Characteristics of the twelve rhythms used in the synchronization tapping tasks.

Trial	Rhythm	Tempo	Random pitch
1	Isochronous	600	No
2	Isochronous	700	No
3	Isochronous	600	Yes
4	Isochronous	700	Yes
5	Strong no. 706	600	No
6	Strong no. 737	700	No
7	Strong no. 706	600	Yes
8	Strong no. 737	700	Yes
9	Weak no. 960	600	No
10	Weak no. 1858	700	No
11	Weak no. 960	600	Yes
12	Weak no. 1858	700	Yes

*Rhythm numbers for strongly and weakly metrical rhythms refer to the different rhythms depicted in **Figure 1**. Tempo orders were counterbalanced between participants.*

### PROCEDURE

Spontaneous tapping data were collected for all participants before they engaged in the synchronization tasks using acoustic stimuli. Participants were asked to tap at a comfortable pace and to make 40 taps (of which the first 10 were excluded from analysis).

In the synchronization tests, the instruction was to tap out a regular beat in synchrony with the rhythmic sequences that were played. This meant that for the sequences with an isochronous beat, participants were required to tap on every auditory event. For the strongly and weakly metrical rhythms in contrast, participants were required to extract the underlying beat and tap in time with this, meaning that not every tap made would align with an auditory event. The twelve different trials as outlined in **Table 1** dissociated possible effects on tapping performance of rhythm type, tempo, and variation in pitch. Participants were required to synchronize to 48 downbeats in each trial (corresponding to 12 cyclical repetitions of the sequences), with recorded responses to the first eight downbeats being discarded from analysis.

Each trial started with the presentation of eight initial beats during which the participant could listen to the stimulus and start tapping along if they wished to, but during which their tapping was not recorded. A timer bar on the screen indicated this familiarization period, at the end of which tapping started to be recorded.

## RESULTS

Scores on the MBEA are given in **Table 2** and demonstrate that dysrhythmic participants were scoring below threshold for rhythm subtests but above threshold for pitch subtests.

**Table 2:** MBEA scores for dysrhythmic participants.

Subject	Scale	Contour	Interval	Rhythm	% Correct pitch (amalgamated)	% Correct rhythm
SS	29	25	24	22	87	73
SWI	28	30	27	21	94	70
VPO	29	26	27	20	82	68

*
Scores below 23 are considered to indicate impairments.
*

### SPONTANEOUS TAPPING TASK

For the spontaneous tapping task, in which participants were asked to tap a regular beat in the absence of an acoustic stimulus, we analyzed the mean tapping rate, and variability in tapping rate for each individual. The first 10 taps were excluded from analysis, in order to given an adequate “lead-in” time for participants to start tapping at a regular rate. The individual mean tapping rate (in ms) was calculated for each participant. The standard deviation of the inter-tap-intervals (ITIs) for each participant was used as a measure of variance (in ms), and this value was divided by the individual’s mean tapping rate for that stimulus to give the coefficient of variation (CV) for spontaneous ITIs.

**FIGURE 2:**
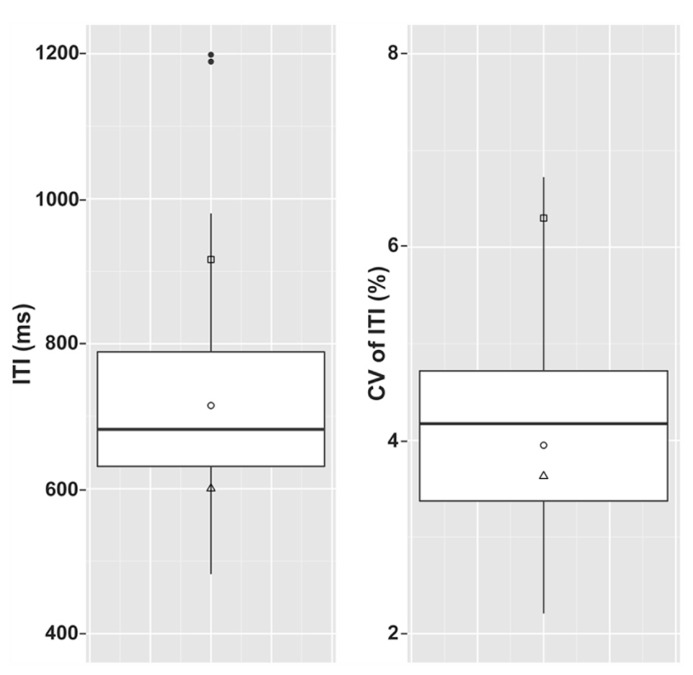
**Inter-tap-intervals (ITIs) and coefficient of variation of ITIs in the three dysrhythmic participants compared to the control group for the spontaneous tapping task.** Boxes summarize the control group data. Top and bottom edges of the boxes give 1st and 3rd quartiles and the middle bands give the group medians. Whiskers include data that is within 1.5 interquartile ranges of the 1st and 3rd quartiles and filled circles represent control data outside of this range. Left panel gives raw ITI values and right panel gives standard deviations of ITI as a percentage of mean spontaneous tapping rate. □ = SS; ∘ = SWI; △ = VPO. Filled circles represent outlying control data.

A summary of spontaneous tapping data from the dysrhythmic individuals compared to the controls is given in **Figure 2**. The dysrhythmics’ data were evaluated in comparison to the control group data (after log transformation) using “singlims.exe,” a program developed by Crawford and Garthwaite (2002) to evaluate single subject values relative to a control group based on Bayesian statistics. The dysrhythmics’ individual mean tapping rates did not significantly differ from those of the controls; all three values were within 1.5 standard deviations of the control group’s mean tapping rate. Variability in tapping rate however, was significantly different from the control group for one participant (SS; *t* = 1.96, *p* = 0.029 one tailed). Both of the other dysrhythmic participants in contrast tapped with a slightly lower average variability in comparison to the controls.

### SYNCHRONIZATION TASKS

**FIGURE 3:**
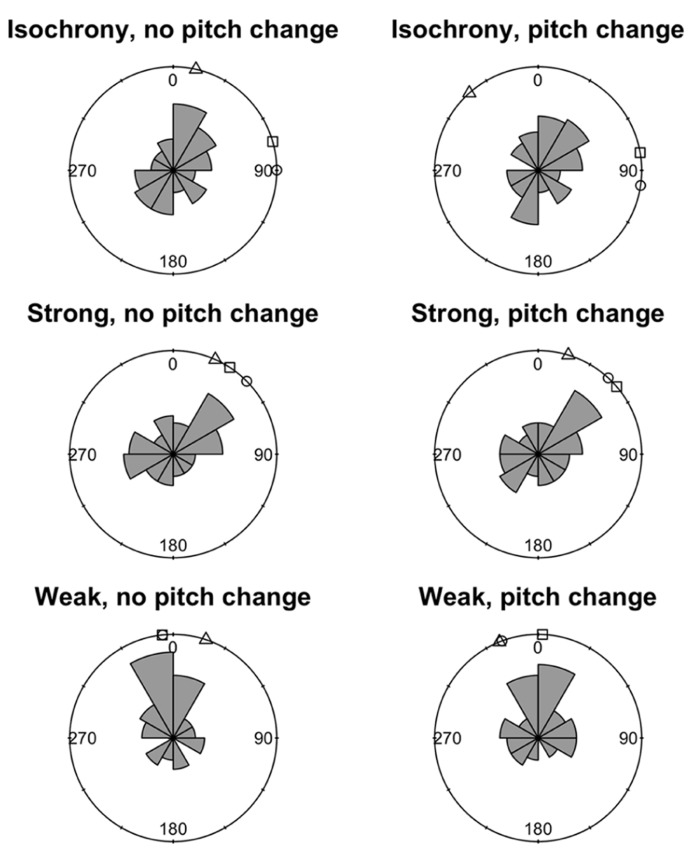
**Mean circular asynchronies for the three dysrhythmic participants compared to the control group in all six conditions of the synchronization task.** Circular histograms depict frequency density of mean asynchronies around a circle. Values close to 0° indicate tapping close to the correct tap time, while values close to 180° indicate tapping halfway between the correct times. □ = SS; ∘ = SWI; △ = VPO.

We used a circular statistics approach to derive the mean asynchrony (a measure of tap time accuracy) of synchronization trials. The onset-asynchrony for each tap was captured as an error value relative to the position of the corresponding downbeat (i.e., how late or early the participant tapped in relation to the 40 downbeat locations in the acoustic stimuli). The individual tap-to-onset-asynchrony was transformed into a circular asynchrony by dividing by the most recent ITI and multiplying by 360. Circular statistics (e.g., Mardia and Jupp, 1999) were then used to calculate the mean asynchrony for each participant in each condition and these are summarized in **Figure 3**. As mean asynchronies in each condition were highly variable even within control participants these were not statistically analyzed in terms of differences between dysrhythmics and controls.

**FIGURE 4:**
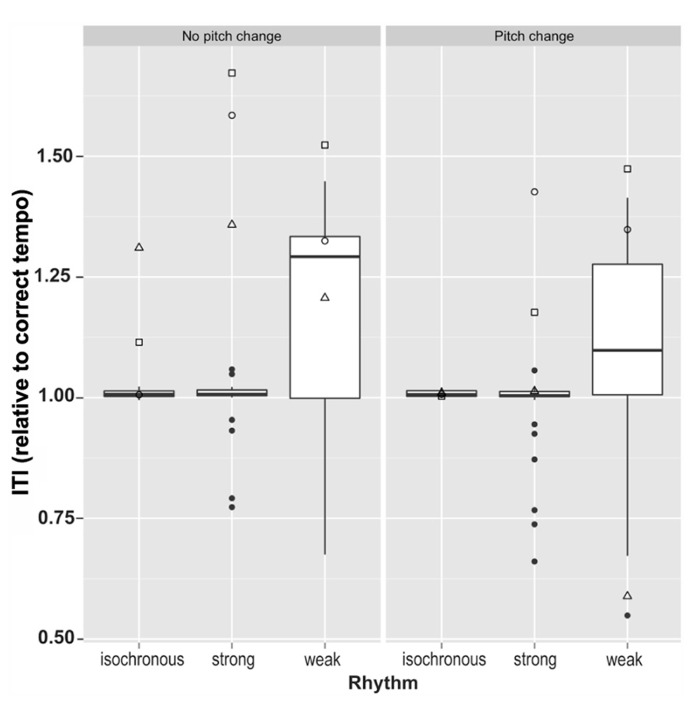
**Mean tapping rates relative to correct tapping rate in all six conditions for the three dysrhythmics compared to the controls.** Here, control data largely lies within very small limits for the isochronous and strongly metrical conditions. □ = SS; ∘ = SWI; △ = VPO.

To assess the ability to tap at the intended rate along to the stimuli we calculated the mean tapping rate and regularity of tapping rate. This was done by first dividing ITIs by the tempo of the trial to give relative tapping rates and enable comparison across the two tempi (excluding all outliers of more than 2.5 standard deviations from the sample mean ITI). One control participant’s data was excluded entirely, due to two trials in which all ITIs were outliers relative to the remaining participants’ data. Mean relative tapping rates for each condition are summarized in **Figure 4**. The data demonstrate that control participants tapped very close to the expected rate in most conditions, although displaying a large amount of variability in the weakly metrical conditions.

**FIGURE 5:**
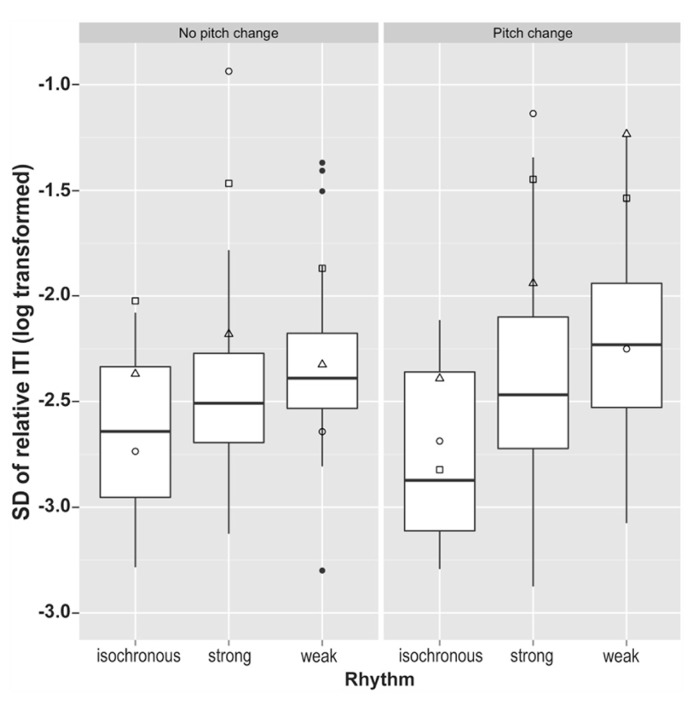
**Variability in tapping rate in all six conditions for the synchronization task.** □ = SS; ∘ = SWI; △ = VPO. Filled circles represent outlying control data.

The standard deviation of relative tapping rate (as calculated above) was used to measure an individual’s tapping stability within a trial. After log transformation in order to obtain normally distributed data samples, control data were compared across different conditions using a 3 (rhythm type: isochronous, strong, weak) × 2 (pitch: random variation, no pitch change) ANOVA, with Greenhouse–Geisser correction for non-sphericity. A significant main effect of rhythm condition was identified, *F*(2,70) = 21, *p* < 0.0001, Gη^2^ = 0.17, no main effect of pitch (*p* = 0.41), and a near significant interaction between the two, *F*(2,70) = 2.97, *p* = 0.058, Gη^2^ = 0.012. *Post hoc* pairwise *t*-tests comparing the three rhythm types, with Bonferroni correction for multiple comparison, demonstrated that variability in tapping rate differed significantly between all three rhythm types. Performance for isochronous rhythms exhibited significantly smaller variability in tapping rate than for both strongly metrical rhythms (*p* < 0.0001) and weakly metrical rhythms (*p* < 0.0001), while strongly metrical rhythms demonstrated significantly lower variability in tapping rate compared to weakly metrical rhythms (*p* = 0.001). These results are summarized in **Figure 5**.

**FIGURE 6:**
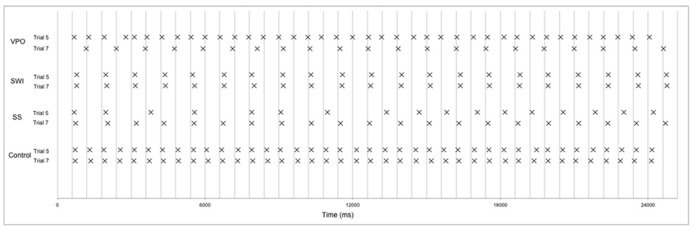
**Raw tapping data in Trial 5 and Trial 7 for dysrhythmics and one control subject (randomly selected).** Lines give the correct tap times, and the midpoint of each × represents an individual tap. Taps corresponding to the first 40 tones are given here.

The dysrhythmics’ synchronization tapping data exhibited a qualitative difference in performance, identifiable at a gross level; while all three dysrhythmics often produced the correct number of taps for the isochronous sequences, they typically produced only half as many taps as there were downbeats for the strongly metrical rhythms. This might indicate that the dysrhythmics were tapping at half the tempo of the intended meter. The number of “missed beats,” i.e., the discrepancy between the 40 presented downbeats in the stimuli and the number of taps produced on each trial, are given in **Table 3**, along with mean ITIs for dysrhythmics compared to controls. The numbers support the notion that dysrhythmics tended to tap at half the expected tempo, which did not occur in the control data. In addition, as can be seen in **Figure 5**, variability in tapping rate for dysrhythmics was higher than in controls in these conditions, which may be a consequence of tapping at a much slower tempo than intended. Owing to this assumed difference in hierarchical level with which the dysrhythmics compared to the control participants synchronized their tapping, we refrained from performing formal statistical analysis on the synchronization accuracy and variability measures. **Figure 6** gives raw tapping data for dysrhythmics compared to one randomly selected control subject in Trials 5 and 7, to demonstrate the dysrhythmics tendency to tap regularly, but at a slower tempo corresponding to the next-higher hierarchical level than intended.

**Table 3:** Number of missed beats and mean ITIs in the dysrhythmic participants compared to the control group.

Measure	Participant	Trial 1	Trial 2	Trial 3	Trial 4	Trial 5	Trial 6	Trial 7	Trial 8	Trial 9	Trial 10	Trial 11	Trial 12
Missed	SS	0	0	9	0	**23**	11	**19**	**19**	*	**17**	13	**17**
taps	SWI	0	0	0	0	**19**	**19**	**19**	**19**	*	10	9	9
	VPO	1	0	0	7	0	1	**19**	0	11	12	6	12
	Control mean	0.05	0.29	0.05	0.11	0.87	1.16	0.87	0.26	6.32	5.97	6.76	6.47
Mean	SS	600	704	770	701	**1294**	389	**1178**	**1366**	*	**1150**	882	**1164**
ITI (ms)	SWI	605	704	604	703	**1189**	**1387**	**1190**	**1386**	*	943	795	926
	VPO	614	713	600	1223	601	717	**1140**	721	337	381	723	386
	Control mean	604	713	606	707	595	678	596	700	718	816	703	818

Numbers in bold indicate that subjects miss out approximately half of the expected beats. *indicates missing data.

In weakly metrical trials, performance was comparably poor for all participants, with beats being “missed” frequently in both participant groups. It is clear from **Figure 4** furthermore, that control participants were not always tapping with the expected tempo. This problem in tapping at the expected tempo is likely to reflect the fact that even controls have problems extracting the meter in these trials thus the poor performance of dysrhythmics in these conditions is unsurprising.

## DISCUSSION

The current study investigates individuals with what we are terming “dysrhythmia”: a congenital, selective deficit in rhythm perception and production. This deficit appears to be much rarer than the form of amusia that has been characterized as a selective pitch impairment. Importantly, the dysrhythmic participants studied here did not demonstrate a general problem with spontaneous tapping, indicating that these individuals are unlikely to suffer from motor deficits that could explain synchronization performance. Their difficulties with tapping along to the beat of different types of rhythm are thus likely to specifically relate to issues with extracting rhythmic information. In the present cases, both rhythm perception and production tasks revealed anomalies relative to the performance of the controls, including both an impairment in musical rhythm perception, measured via the MBEA, and abnormal tapping behavior when required to extract the beat from a rhythmic sequence.

If beat-based rhythm production was generally impaired in dysrhythmic subjects then we would expect to also find difficulties in the maintenance of a self-paced steady beat. However, no difference in spontaneous tapping rate was found between control subjects and the dysrhythmics, apart from slightly larger variability in tapping rate for one dysrhythmic compared to the controls. Overall, the normal ability of these individuals to produce a self-paced steady beat demonstrates that rhythmic difficulties cannot be ascribed to motor deficits with generating and maintaining a steady beat. One might expect, therefore, that dysrhythmics would be able to perform normally if required to continue tapping out an isochronous beat after entraining with an acoustic stimulus. Their problem seems to lie specifically in extracting the correct (intended) meter from non-isochronous metrical rhythms. This differentiation merits future investigation.

Consistent with the notion of a close-to normal ability to produce a steady beat, basic sensorimotor entrainment was largely preserved: for synchronization with an isochronous beat, tapping rates in dysrhythmic participants (**Table 3**) demonstrate that they were mostly tapping at the intended tempo, as expected in normal participants and seen in the controls. When required to extract a metrical-beat and synchronize to it, in contrast, the dysrhythmics’ tapping behavior deviated significantly from that of the controls. For the strongly metrical sequences, the dysrhythmics produced approximately half of the beats required for the intended downbeat, suggesting that they had difficulties extracting a regular beat from a rhythm (something controls can do with ease). In weakly metrical conditions all participants performed badly, suggesting a floor effect for these rhythm sequences.

The finding of entraining at an unexpected metrical level is very similar to that of Dalla Bella and Peretz (2003) who studied congenitally amusic subjects with pitch-based deficits, and this similarity suggests that there may be some patterns of impairment that generalize between these groups. Entraining at a higher metrical level can occur because subjective grouping of tones in metrical sequences is possible in a number of ways depending on which level of the metrical-beat hierarchy one perceives as the most prominent and comfortable one. For a strongly metrical rhythm such as in the sequences used here, up to three potential levels of perception of the beat exist: one at half, one at twice, and one at the tempo of the intended dominant meter. According to behavioral literature of the past decades promoting a range from a minimum of 200 to up to about 1000 ms (e.g., see Repp, 2005), people would generally entrain at the intended meter of 600 or 700 ms. However, humans tend to be quite capable of tapping along in time to music at a range of tempos, and the criteria used to evaluate this ability in musically oriented work has typically been based on whether successive taps coincide with beats in the music, regardless of the hierarchical level this occurs at (Drake and Bertrand, 2001). Whether the current finding is due to a rate-based limitation (i.e., slowing-down of the processing of metrical periodicity) or a deficit in multi-level hierarchical processing of metrical structure remains to be explored.

Either way, this problem is quite different from “poor synchronizers” as identified by Sowinski and Dalla Bella (2013), who exhibit poor synchronization as a consequence of error correction difficulties. Participants in their study did not have problems with rhythm perception, although some may have experienced synchronization problems as a consequence of poor pitch perception. The heterogeneity of synchronization disorders point to the complexity of the processes underlying this ability, and suggests that much further work is required to find out how these problems arise.

To our knowledge, this is the first report of a type of musical deficit that demonstrates impaired rhythm perception and beat extraction in the face of intact pitch perception, in neurologically intact individuals. This pattern is the opposite of that which has previously been reported in individuals termed congenitally amusic, according to the MBEA, where as many as half are reported to have pitch deficits in the face of normal scores on the rhythm test (Ayotte et al., 2002). We suggest that the findings from these individuals contribute to an emerging picture of the different ways in which rhythm perception can be compromised, with theoretical implications for our understanding of the processing of rhythm in the typical population.

## Conflict of Interest Statement

The authors declare that the research was conducted in the absence of any commercial or financial relationships that could be construed as a potential conflict of interest.

## References

[B1] Ayotte J. Peretz I. Hyde K. (2002). Congenital amusia a group study of adults afflicted with a music-specific disorder. *Brain* 125 238–251 10.1093/brain/awf028 11844725

[B2] Beebe B. Lachmann F. M. (1988). The contribution of mother-infant mutual influence to the origins of self-and object representations. *Psychoanal. Psychol.* 5 305–337 10.1037/0736-9735.5.4.305

[B3] Bernieri F. J. Reznick J. S. Rosenthal R. (1988). Synchrony, pseudosynchrony, and dissynchrony: measuring the entrainment process in mother-infant interactions. *J. Pers. Soc. Psychol.* 54 243–253 10.1037/0022-3514.54.2.243

[B4] Crawford J. R. Garthwaite P. H. (2002). Investigation of the single case in neuropsychology: confidence limits on the abnormality of test scores and test score differences. *Neuropsychologia* 40 1196–1208 10.1016/S0028-3932(01)00224-X 11931923

[B5] Dalla Bella S. Peretz I. (2003). Congenital amusia interferes with the ability to synchronize with music. *Ann. N. Y. Acad. Sci.* 999 166–169 10.1196/annals.1284.021 14681133

[B6] Dissanayake E. (2000). “Antecedents of the temporal arts in early mother-infant interaction,” in *The Origins of Music,* eds Wallin N. L. Merker B. R. Brown S. (Cambridge, Mass.: MIT Press) 389–410

[B7] Drake C. Bertrand D. (2001). The quest for universals in temporal processing in music. *Ann. N. Y. Acad. Sci.* 930 17–27 10.1111/j.1749-6632.2001.tb05722.x 11458828

[B8] Ehrle N. Samson S. (2005). Auditory discrimination of anisochrony: influence of the tempo and musical backgrounds of listeners. *Brain Cogn.* 58 133–147 10.1016/j.bandc.2004.09.014 15878734

[B9] Farrugia N. Benoit C. E. Harding E. Kotz S. A Dalla Bella S. (2012). BAASTA: battery for the assessment of auditory sensorimotor and timing abilities. *Inst. Hum. Cogn. Brain Sci.* 18 9 10.3758/s13428-016-0773-6 27443353

[B10] Foxton J. M. Nandy R. K. Griffiths T. D. (2006). Rhythm deficits in “Tone deafness”. *Brain Cogn.* 62 24–29 10.1016/j.bandc.2006.03.005 16684584

[B11] Fraisse P. (1963). *The Psychology of Time*. Oxford, UK: Harper & Row

[B12] Friberg A. Sundberg J. (1995). Time discrimination in a monotonic, isochronous sequence. *J. Acoust. Soc. Am.* 98 2524–2531 10.1121/1.413218

[B13] Grube M. Griffiths T. D. (2009). Metricality-enhanced temporal encoding and the subjective perception of rhythmic sequences. *Cortex* 45 72–79 10.1016/j.cortex.2008.01.006 19058797

[B14] Grube M. Kumar S. Cooper F. E. Turton S. Griffiths T. D. (2012). Auditory sequence analysis and phonological skill. *Proc. R. Soc. B Biol. Sci.* 279 4496–4504 10.1098/rspb.2012.1817 PMC3479813 22951739

[B15] Handel S. (1998). The interplay between metric and figural rhythmic organization. *J. Exp. Psychol. Hum. Percept. Perform.* 24 1546–1561 10.1037/0096-1523.24.5.1546

[B16] Hannon E. E. Trehub S. E. (2005). Tuning in to musical rhythms: infants learn more readily than adults. *Proc. Natl. Acad. Sci. U.S.A.* 102 12639–12643 10.1073/pnas.0504254102 16105946 PMC1194930

[B17] Hebert S. Cuddy L. L. (2002). Detection of metric structure in auditory figural patterns. *Percept. Psychophys.* 64 909–918 10.3758/BF03196795 12269298

[B18] Hirsh I. J. Monahan C. B. Grant K. W. Singh P. G. (1990). Studies in auditory timing: 1. Simple patterns. *Percept. Psychophys.* 47 215–226 10.3758/BF03204997 2326145

[B19] Hove M. J. Risen J. L. (2009). It’s all in the timing: interpersonal synchrony increases affiliation. *Soc. Cogn.* 27 949–960 10.1521/soco.2009.27.6.949

[B20] Huss M. Verney J. P. Fosker T. Mead N. Goswami U. (2011). Music, rhythm, rise time perception and developmental dyslexia: perception of musical meter predicts reading and phonology. *Cortex* 47 674–689 10.1016/j.cortex.2010.07.010 20843509

[B21] Hyde K. L. Peretz I. (2004). Brains that are out of tune but in time. *Psychol. Sci.* 15 356–360 10.1111/j.0956-7976.2004.00683.x% 15102148

[B22] Iversen J. R. Patel A. D. (2008). “The Beat Alignment Test (BAT): surveying beat processing abilities in the general population,” in *Proceedings of the 10{^th^ International Conference on Music Perception and Cognition (ICMPC10)*} (Sapporo: Casual Productions) 465–468

[B23] Jusczyk P. W. Hirsh-Pasek K. Nelson D. G. Kennedy L. J. Woodward A. Piwoz J. (1992). Perception of acoustic correlates of major phrasal units by young infants. *Cogn. Psychol.* 24 252–293 10.1016/0010-0285(92)90009-Q 1582173

[B24] Jusczyk P. W. Houston D. M. Newsome M. (1999). The beginnings of word segmentation in English-learning infants. *Cogn. Psychol.* 39 159–207 10.1006/cogp.1999.0716 10631011

[B25] Knoblich G. Butterfill S. Sebanz N. (2011). Psychological research on joint action: theory and data. *Psychol. Learn. Motiv.* 54 59–101 10.1016/B978-0-12-385527-5.00003-6

[B26] Konvalinka I. Xygalatas D. Bulbulia J. Schj{ø}dt U. Jegind{ø} E. M. Wallot S. (2012). Synchronized arousal between performers and related spectators in a fire-walking ritual. *Proc. Natl. Acad. Sci.* 108 8514–8519 10.1073/pnas.1016955108 21536887 PMC3100954

[B27] Launay J. Dean R. T. Bailes F. (2013). Synchronization can influence trust following virtual interaction. *Exp. Psychol.* 60 53–63 10.1027/1618-3169/a000173 22935329

[B28] London J. (2004). *Hearing in Time*. New York: Oxford University Press 10.1093/acprof:oso/9780195160819.001.0001

[B29] Mardia K. V. Jupp P. E. (1999). *Directional Statistics*. Chichester: Wiley 10.1002/9780470316979

[B30] Monahan C. B. Hirsh I. J. (1990). Studies in auditory timing: 2. Rhythm patterns. *Percept. Psychophys.* 47 227–242 10.3758/BF03204998 2326146

[B31] Oullier O. De Guzman G. C. Jantzen K. J. Lagarde J Kelso J. A. S. (2008). Social coordination dynamics: measuring human bonding. *Soc. Neurosci.* 3 178–192 10.1080/17470910701563392 18552971 PMC2156197

[B32] Patel A. D. Iversen J. R. Chen Y. Repp B. H. (2005). The influence of metricality and modality on synchronization with a beat. *Exp. Brain Res.* 163 226–238 10.1007/s00221-004-2159-8 15654589

[B33] Peretz I. Champod A. S. Hyde K. (2003). Varieties of musical disorders. *Ann. N. Y. Acad. Sci.* 999 58–75 10.1196/annals.1284.006 14681118

[B34] Peretz I. Hyde K. L. (2003). What is specific to music processing? Insights from congenital amusia. *Trends Cogn. Sci.* 7 362–367 10.1016/S1364-6613(03)00150-5 12907232

[B35] Phillips-Silver J. Toiviainen P. Gosselin N. PichÈ O. Nozaradan S. Palmer C. (2011). Born to dance but beat deaf: a new form of congenital amusia. *Neuropsychologia* 49 961–969 10.1016/j.neuropsychologia.2011.02.002 21316375

[B36] Phillips-Silver J. Trainor L. J. (2005). Feeling the beat: movement influences infant rhythm perception. *Science* 308 1430–1430 10.1126/science.1110922 15933193

[B37] Povel D. J. Essens P. (1985). Perception of temporal patterns. *Music Percept.* 2 411–440 10.2307/40285311 3991313

[B38] Praamstra P. Turgeon M. Hesse C. Wing A. Perryer L. (2003). Neurophysiological correlates of error correction in sensorimotor-synchronization. *Neuroimage* 20 1283–1297 10.1016/S1053-8119(03)00351-3 14568497

[B39] Repp B. H. (2005). Sensorimotor synchronization: a review of the tapping literature. *Psychon. Bull. Rev.* 12 969–992 10.3758/BF03206433 16615317

[B40] Smith M. R. Cutler A. Butterfield S. Nimmo-Smith I. (1989). The perception of rhythm and word boundaries in noise-masked speech. *J. Speech Hear. Res.* 32 912–920 2601320 10.1044/jshr.3204.912

[B41] Sowinski J Dalla Bella S. (2013). Poor synchronization to the beat may result from deficient auditory-motor mapping. *Neuropsychologia* 51 1952–1963 10.1016/j.neuropsychologia.2013.06.027 23838002

[B42] ten Hoopen G. Boelaarts L. Gruisen A. Apon I. Donders K. Mul N. (1994). The detection of anisochrony in monaural and interaural sound sequences. *Percept. Psychophys.* 56 110–120 10.3758/BF03211694 8084727

[B43] Thaut M. H. Abiru M. (2010). Rhythmic auditory stimulation in rehabilitation of movement disorders: a review of the current research. *Music Percept. Interdiscip. J.* 27 263–269 10.1525/mp.2010.27.4.263

[B44] Vickhoff B. Malmgren H. Åström R. Nyberg G. Ekström S.-R. Engwall M. (2013). Music structure determines heart rate variability of singers. *Front. Psychol.* 4:334. 10.3389/fpsyg.2013.00334 PMC3705176 23847555

[B45] Wiltermuth S. S. Heath C. (2009). Synchrony and cooperation. *Psychol. Sci.* 20 1–5 10.1111/j.1467-9280.2008.02253.x 19152536

[B46] Wing A. Kristofferson A. (1973). Response delays and the timing of discrete motor responses. *Percept. Psychophys.* 14 5–12 10.3758/BF03198607

[B47] Zentner M. Eerola T. (2010). Rhythmic engagement with music in infancy. *Proc. Natl. Acad. Sci.* 107 5768–5773 10.1073/pnas.1000121107 20231438 PMC2851927

