# Corrigendum: Dysrhythmia: a specific congenital rhythm perception deficit

**DOI:** 10.3389/fpsyg.2014.01077

**Published:** 2014-09-26

**Authors:** Lauren Stewart

**Affiliations:** Music, Mind and Brain, Department of Psychology, Goldsmiths University of LondonLondon, UK

**Keywords:** rhythm, meter, beat, motor timing, amusia

This study was supported by a grant to Lauren Stewart from the Economic and Social Research council (ESRC; RES-061-25-0155).

## Conflict of interest statement

The author declares that the research was conducted in the absence of any commercial or financial relationships that could be construed as a potential conflict of interest.

